# Nasal high-flow oxygen therapy system for improving sleep-related hypoventilation in chronic obstructive pulmonary disease: a case report

**DOI:** 10.1186/1752-1947-8-341

**Published:** 2014-10-13

**Authors:** Miyuki Okuda, Makoto Kashio, Nobuya Tanaka, Tomoshige Matsumoto, Sumiko Ishihara, Tatsuo Nozoe, Takashi Fujii, Yoshinari Okuda, Toshiomi Kawahara, Keigo Miyata

**Affiliations:** 1 Osaka Hospital, Internal Medicine Department, Neyagawakoen 2276-1, Neyagawa City, Osaka 572-0854, Japan; 2 Osaka Hospital, Surgery Department, Neyagawakoen 2276-1, Neyagawa City, Osaka 572-0854, Japan; 3 Osaka Hospital, Nursing Department, Neyagawakoen 2276-1, Neyagawa City, Osaka 572-0854, Japan; 4 Osaka Hospital, Rehabilitation Department, Neyagawakoen 2276-1, Neyagawa City, Osaka 572-0854, Japan; 5 Okuda Clinic, Internal Medicine, Uchiage1123, Neyagawa, Osaka 572-0858, Japan; 6 Shinsei Company Limited, Minamiterakatahigashidori 2-9-1, Moriguchi, Osaka 570-0043, Japan; 7 Chest Company Limited, Hongo 3-25-11, Bunkyoku, Tokyo 113-0033, Japan

**Keywords:** Nasal high-flow oxygen therapy, Sleep-related hypoventilation, Chronic obstructive pulmonary disease

## Abstract

**Introduction:**

Sleep-related hypoventilation should be considered in patients with chronic obstructive pulmonary disease, because appropriate respiratory management during sleep is important for preventing elevation of PaCO_2_ levels. A nasal high-flow oxygen therapy system using a special nasal cannula can deliver suitably heated and humidified oxygen at up to 60 L/min. Since the oxygen concentration remains a constant independent of minute ventilation, this system is particularly useful in patients with chronic obstructive pulmonary disease who have hypercapnia. This is the first report of sleep-related hypoventilation with chronic obstructive pulmonary disease improving using a nasal high-flow oxygen therapy system.

**Case presentation:**

We report the case of a 73-year-old Japanese female who started noninvasive positive-pressure ventilation for acute exacerbation of chronic obstructive pulmonary disease and CO_2_ narcosis due to respiratory infection. Since she became agitated as her level of consciousness improved, she was switched to a nasal high-flow oxygen therapy system. When a repeat polysomnography was performed while using the nasal high-flow oxygen therapy system, the Apnea Hypopnea Index was 3.7 times/h, her mean SpO_2_ had increased from 89 to 93%, percentage time with SpO_2_ ≤ 90% had decreased dramatically from 30.8 to 2.5%, and sleep stage 4 was now detected for 38.5 minutes. As these findings indicated marked improvements in sleep-related hypoventilation, nasal high-flow oxygen therapy was continued at home. She has since experienced no recurrences of CO_2_ narcosis and has been able to continue home treatment.

**Conclusions:**

Use of a nasal high-flow oxygen therapy system proved effective in delivering a prescribed concentration of oxygen from the time of acute exacerbation until returning home in a patient with chronic obstructive pulmonary disease, dementia and sleep-related hypoventilation. The nasal high-flow oxygen therapy system is currently used as a device to administer high concentrations of oxygen in many patients with type I respiratory failure, but may also be useful instead of a Venturi mask in patients like ours with type II respiratory failure, additionally providing some positive end-expiratory pressure.

## Introduction

Sleep-related hypoventilation (SRH) should be considered in patients with chronic obstructive pulmonary disease (COPD), because appropriate respiratory management during sleep is important in preventing elevations in PaCO_2_ levels. Variations in minute ventilation must be considered in the respiratory management of COPD, and the oxygen delivery system used should be capable of high flow rates in which fractional inspired oxygen concentrations (FiO_2_) do not vary with the patient’s breathing pattern. We have previously reported that the average volume-assured pressure support (AVAPS) ventilatory mode was useful in achieving target minute ventilation in a patient with acute exacerbation of COPD who had sleep-disordered breathing.

We report herein the case of a patient in whom a nasal high-flow oxygen therapy system (NHFOTS; Pacific-Medico, Tokyo, Japan) and a biphasic positive airway pressure (BiPAP) machine (Vivo 30; Breas Medical AB, Mölnlycke, Sweden) were used for acute management of COPD. The patient was able to be weaned from non-invasive positive-pressure ventilation (NPPV), achieved improvements in SRH, and was subsequently able to be treated at home.

## Case presentation

A 73-year-old Japanese female was being treated by a local physician for hypertension, depression, dementia and insomnia. She developed flu-like symptoms in December 2013, and was diagnosed with influenza/cold by her physician. Clarithromycin (CAM) was prescribed. The next day, her level of consciousness decreased, so she was transported to an urgent care facility. Marked hypoxemia and hypercapnia were then identified, and she was transferred to our hospital for further evaluation and treatment.

She had an axillary temperature of 37.2°C, a heart rate of 82 beats/min and regular, a respiratory rate of 14 breaths/min (tachypneic), a blood pressure of 156/86mmHg, and peripheral oxygen saturation (SpO_2_) of 79% (with nasal oxygen at 2L/min). Her level of consciousness was decreased (Glasgow Coma Scale: eye opening 4, verbal response 3, motor response 5, total 12), but no pallor or icterus was evident.

Her radiography revealed cardiomegaly and increased transparency of the upper lung fields bilaterally, indicating emphysema. Her echocardiography revealed a mild decrease in left ventricular ejection fraction (56%), right atrial and ventricular enlargement, and an estimated pulmonary systolic blood pressure of 57mmHg, indicating moderate pulmonary hypertension.

Respiratory management for type II respiratory failure was started with NPPV (ST mode; inspiratory positive airway pressure 10cmH_2_O; expiratory positive airway pressure 4cmH_2_O; ventilation rate 18 breaths/min; FiO_2_ 30%). For right-sided heart failure with pulmonary hypertension (clinical scenario, CS5), diuretics and a nitrite were started. Hypercapnia gradually improved, but as her level of consciousness improved, she became more agitated and delirious due to dementia, and management by mask became impossible. She was therefore switched to a NHFOTS (oxygen, 5L/min; continuous positive airway pressure (CPAP), 8cmH_2_O).

No recurrence of CO_2_ narcosis was seen subsequently, and hypoxemia improved (SpO_2_, 94-96%). Using the NHFOTS with a high flow rate, respiratory management was able to be continued without any further agitation. Her arterial blood gases (ABGs) were: pH 7.47, PaO_2_ 57mmHg, PaCO_2_ 62mmHg, and HCO_3_^-^ 41.5mmol/L.

Investigation of SRH by polysomnography (PSG) showed an apnea-hypopnea index (AHI) of 12.1 events/h, and SRH was therefore diagnosed. When a repeat PSG was performed while using the NHFOTS, her AHI was 3.7 events/h, her mean SpO_2_ had increased from 89 to 93%, the percentage time with SpO_2_ ≤90% had decreased dramatically from 30.8 to 2.5%, and sleep stage 4 (deep sleep), which previously had not been observed, was now detected for 38.5 minutes. These results indicated marked improvements in SRH (Table 1).

**Table 1:** Polysomnography data for before nasal high-flow therapy system and nasal high-flow therapy system

	**Before NHFOTS**	**NHFOTS**
AHI (events/h)	12.1	3.7
AI (events/h)	0.26	0.0
HI (events/h)	11.9	3.7
Mean SpO_2_ (%)	89	93.0
Minimum SpO_2_ (%)	63	79
CT90 (%)	30.8	2.5
Sleep Stage 1 (min)	109.5	65.0
Sleep Stage 2 (min)	210.5	268.0
Sleep Stage 3 (min)	68.0	84.0
Sleep Stage 4 (min)	0.0	38.5
Sleep Stage REM (min)	72.0	85.5

NHFOTS: nasal high-flow therapy system.

AHI: apnea-hypopnea index.

AI: apnea index.

HI: hypopnea index.

SpO_2_: Oxygen Saturation of Arterial Blood Measured by Pulse Oximeter.

CT90: cumulative time percentage with SpO2 <90%.

REM: Rapid eye movement.

Pulmonary function testing revealed severe obstructive ventilatory impairment and decreased pulmonary diffusion capacity. Based on the above test results, her classification as grade 3 on the modified Medical Research Council scale for exertional dyspnea, and her decreased percentage-predicted forced expiratory volume in one second, she was diagnosed with category D COPD, based on the Global Initiative for Chronic Obstructive Lung Disease (GOLD) guidelines, and also with SRH. She was discharged, and NHFOTS was started at home. As of the time of writing, she is continuing treatment with the NHFOTS at home, has shown no increases in PaCO_2_ (her ABGs as of: pH 7.46, PaO_2_ 85mmHg, PaCO_2_ 41mmHg, HCO_3_^-^ 29.2mmol/L), and is being followed up on.

## Discussion

A wide range of evidence has shown that NPPV offers an effective treatment for acute exacerbations of COPD [1-3]. The GOLD guidelines also recommend NPPV, since this method helps to avoid intubation, removes the risk of nosocomial infections such as pneumonia during intubated mechanical ventilation, and is effective in reducing mortality rates [4]. In addition, we have previously reported that the AVAPS mode was useful in achieving target minute ventilation in a patient with acute exacerbation of COPD who had sleep-disordered breathing [5]. However, a key disadvantage of NPPV is that the method is highly reliant on patient cooperation.

Our patient had dementia due to sequelae from a previous subarachnoid hemorrhage and stroke, as well as agitation, depression and disturbance of sleep. She was taking medications prescribed by her previous physician including quetiapine, triazolam, flunitrazepam, etizolam, sulpiride and duloxetine. In such patients, continuous NPPV can be difficult, and use of a NHFOTS with a nasal cannula rather than a face mask can prove effective. We therefore switched ventilatory support devices. The American Thoracic Society states that nasal high-flow oxygen therapy using suitably warmed and humidified oxygen represents a second-line option for oxygen delivery. The NHFOTS uses a special nasal cannula and heated humidifier and can deliver oxygen at maximum flow rates of 30 to 60L/min. Appropriate heating and humidification (37°C, 100% relative humidity) significantly enhances mucociliary clearance without causing nasal discomfort [6].

**Figure 1:**
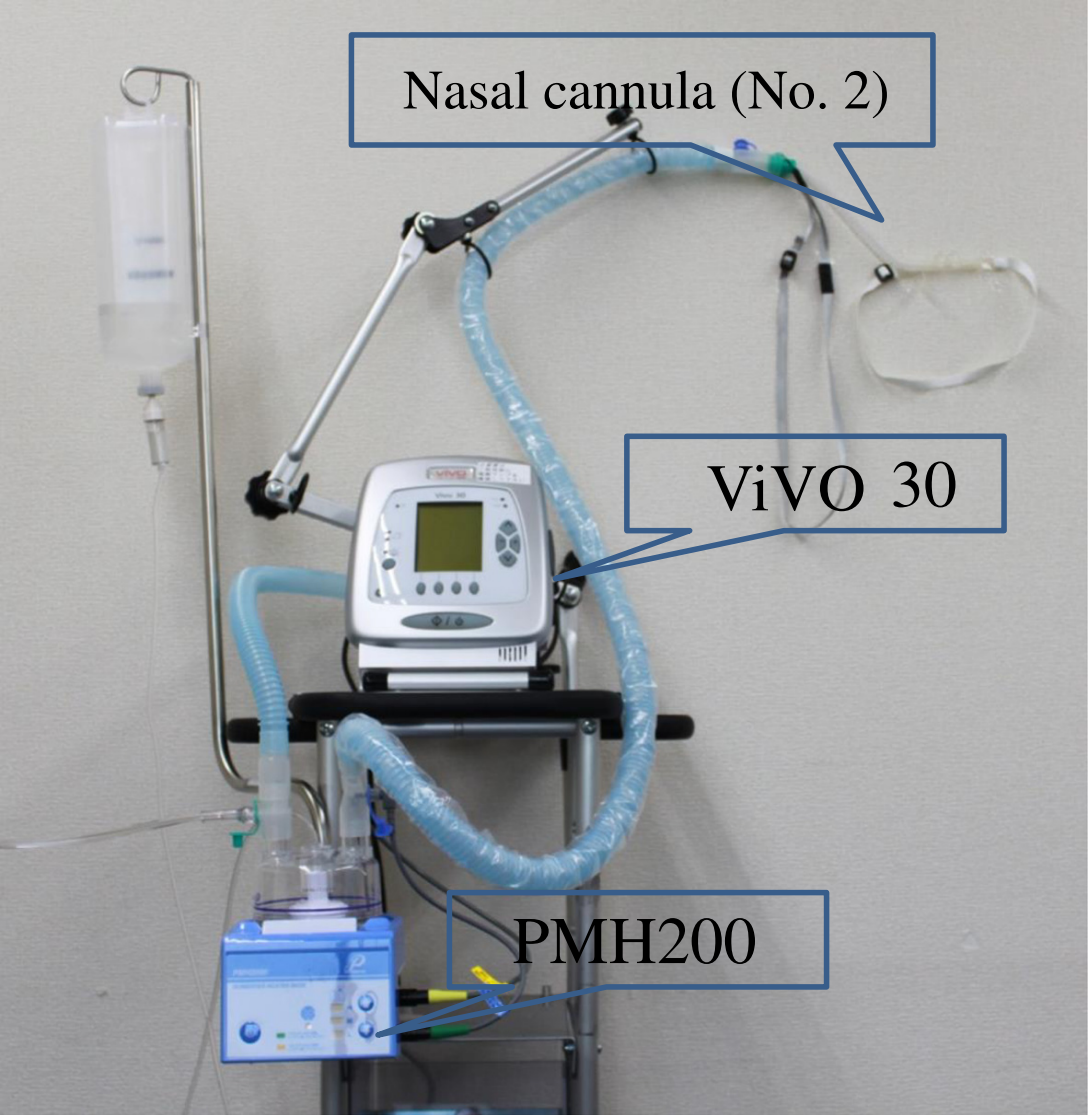
**Equipment used for the nasal high-flow oxygen therapy system.** We used a PMH-2000 heated humidifier, number 2 nasal cannula and Vivo 30 ventilator, which uses a blower assembly to deliver high flow rates.

In our patient, we used a PMH-2000 heated humidifier (Pacific-Medico), a number 2 nasal cannula, and a Vivo 30 ventilator (Breas Medical AB), which uses a blower assembly to deliver high flow rates (Figure 1). This system enables nasal delivery of oxygen at high concentrations and high flow rates. The advantages of a NHFOTS are that patients can still eat and converse, oral care is possible, there is less of an oppressive feeling compared to wearing a mask, and patient quality of life is improved [7]. In our patient with dementia and agitation NHFOTS, unlike NPPV, was able to be continued. Nasal high-flow oxygen therapy, mainly using a nasal high-flow cannula (Optiflow™; Fisher & Paykel Healthcare, Auckland, New Zealand), is used worldwide and has frequently been reported in the literature.

Parke *et al.* compared a NHFOTS and a face mask at an oxygen flow rate of 35L/min in 15 patients after cardiac surgery [8]. They reported that with the mouth closed, mean airway pressure was higher with the NHFOTS (2.7cmH_2_O) than with the face mask (0.2cmH_2_O) [8]. A nasal high-flow cannula, compared to other oxygen delivery devices to date, can deliver nasal oxygen at very high flow rates of up to 60L/min.

**Figure 2:**
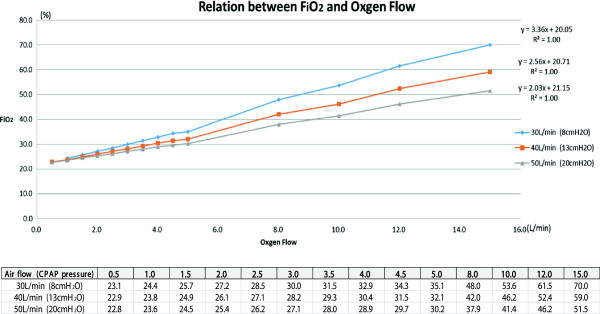
**Relationship between FiO**_**2 **_**and oxygen flow.** CPAP, continuous positive airway pressure.

Figure 2 shows the relationship between different oxygen flow rates and FiO_2_ with the NHFOTS we used. With an oxygen flow rate of 30L/min, when oxygen is supplied at 1L/min, the FiO_2_ is 24.4%. This is equivalent to using a 24% Venturi mask. Although greater leakage is seen with this device, since a pressure of 8cmH_2_O is applied, some PEEP (positive end-expiratory pressure) effect is also seen in comparison to a Venturi mask. When 2.5L/min of oxygen is supplied, the FiO_2_ is 28.5%, similar to that with a 28% Venturi mask. In England, the guidelines for oxygen therapy in acute COPD patients recommend using a Venturi mask, in which by setting the maximum inspiratory flow above the tidal volume of the patient, an accurate oxygen concentration can be delivered independent of the patient’s minute ventilation [9].

With Venturi masks, many types of devices are needed, depending on the oxygen concentration delivered and due to the need for high flow rates. As a result, use of these masks is associated with several disadvantages, including high noise levels, facial and eye irritation, impaired ability to speak and inconvenience for patients when eating. A NHFOTS, by compensating for these disadvantages and using a high-flow oxygen blender, can deliver oxygen at high flow rates up to 60L/min. With nasal high-flow oxygen therapy using a Vivo ventilator, by supplying oxygen at a maximum flow of 15L/min, a 70% oxygen concentration can be achieved, even higher than with a conventional Venturi mask (Figure 2). By increasing CPAP to 8, 13 or 20cmH_2_O, oxygen flow rates can be increased to 30, 40 and 50L/min, respectively. Therefore, unlike a Venturi mask, a NHFOTS can even be used in patients with high minute volumes, such as those who have interstitial lung disease. It is important to understand that by increasing CPAP, the oxygen concentration is decreased even at the same oxygen flow rate (Figure 2). Specifically, for every 1-L/min increase in oxygen, oxygen concentration increases by 3.36% (30L/min), 2.56% (40L/min) and 2.03% (50L/min).

The results from the PSG in our patient confirmed the presence of sleep apnea syndrome (SAS) with an AHI of 12.1 events/h. This type of SRH can be dramatically improved using a NHFOTS during sleep (Table 1), because although the oxygen concentration is low with oxygen supplied at 1.5L/min, the high flow system has a supply flow rate ≥30L/min that, by exerting positive pressure, improves sleep-disordered breathing.

Many factors are involved in the mechanisms of sleep-related alveolar hypoventilation in COPD [10]. Since breathing in COPD patients is highly dependent on accessory muscles of respiration other than the diaphragm, decreased muscle tone during rapid eye movement (REM) sleep increases alveolar hypoventilation. In addition, factors such as decreased functional reserve capacity, increased upper airway resistance and worsening ventilation-perfusion mismatch result in marked hypoxemia, particularly during REM sleep. This sleep hypoxemia increases pulmonary artery pressure, worsening right-sided heart failure [11]. Furthermore, arrhythmias and polycythemia may develop, increasing the mortality rate [12].

Home NPPV is indicated in patients like ours with a PaCO_2_ ≥55mmHg, persistent nocturnal hypoventilation and cor pulmonale. However, if sleep hypoxemia is present, injudicious oxygen administration without taking SRH into consideration can lead to marked hypercapnia. COPD patients, especially those with pulmonary hypertension, should therefore undergo measurement of nighttime oxygen saturation. If oxygen saturation is decreased, PSG should be performed and an appropriate level of oxygen should be prescribed. Regarding acute exacerbations, Austin *et al.* conducted a cluster randomized parallel-group trial in 405 patients with presumed acute exacerbation of COPD during ambulance transport, to compare high-flow oxygen therapy with titrated oxygen therapy [12]. The mortality rate was significantly decreased in the titrated oxygen group (4 versus 9%), and patients in this group with confirmed COPD showed significantly less respiratory acidosis and hypercarbia [12]. With injudicious oxygen administration during sleep, this type of event is likely to recur at night.

Nasal high-flow oxygen therapy essentially requires an oxygen blender. In some settings, such as hospital wards or homes without air supply pipes, an oxygen blender using a Venturi method that can be used with oxygen tubing alone is necessary. This type of set up and oxygen supply is complex. For our patient, we used a modified nasal cannula and PMH-2000 heated humidifier (Pacifico-Medico) and a Vivo 30 ventilator (Breas Medical AB), without using the Venturi method. This system is quiet and convenient for home use. Continued use at home is possible even in patients with dementia, such as our patient, allowing improvements in PaCO_2_.

The disadvantage of a NHFOTS is that ventilatory support is not as effective as NPPV. Reports to date have therefore recommended consideration of NPPV or intubated mechanical ventilation if the respiratory rate and oxygenation have not improved within 4 to 6 hours. Further studies evaluating the use of NPPV for respiratory failure in COPD patients involving a larger number of patients are needed [13,14].

## Conclusion

The use of a NHFOTS was effective for delivering a prescribed concentration of oxygen from the time of acute exacerbation until discharge to home in our patient with COPD who also had dementia and SRH. This safe oxygen therapy enabled improvements in sleep-related alveolar hypoventilation. The NHFOTS is currently used as a device to administer high concentrations of oxygen in many patients with type I respiratory failure, but may also be useful instead of a Venturi mask in patients like ours with type II respiratory failure, in addition to providing some PEEP effect.

## Consent

Written informed consent was obtained from the patient for publication of this case report and any accompanying images. A copy of the written consent is available for review by the Editor-in-Chief of this journal.

## Abbreviations

AHI: Apnea-hypopnea index; AVAPS: Average volume-assured pressure support; COPD: Chronic obstructive pulmonary disease; NHFOTS: Nasal high-flow oxygen therapy system; NPPV: Non-invasive positive pressure ventilation; PSG: Polysomnography; SAS: Sleep apnea syndrome; CPAP: Continuous positive airway pressure; REM: Rapid eye movement; PEEP: positive end-expiratory pressure.

## Competing interests

The authors declare that we have no competing interests.

## Authors’ contributions

MO, SI, TN, TK, and KM gathered the information for this case and were major contributors to the writing of the manuscript. YO, MK, NT, TM and TF contributed to writing the ‘Discussion’ section and editing the manuscript. All authors read and approved the final version of the manuscript.
